# EEG Changes in Migraine—Can EEG Help to Monitor Attack Susceptibility?

**DOI:** 10.3390/brainsci14050508

**Published:** 2024-05-17

**Authors:** Thomas C. van den Hoek, Mark van de Ruit, Gisela M. Terwindt, Else A. Tolner

**Affiliations:** 1 Department of Neurology, Leiden University Medical Centre, 2333 ZA Leiden, The Netherlandsm.l.vanderuit-1@tudelft.nl (M.v.d.R.); g.m.terwindt@lumc.nl (G.M.T.); 2 Department of Biomechanical Engineering, Delft University of Technology, 2628 CD Delft, The Netherlands; 3 Department of Human Genetics, Leiden University Medical Centre, 2300 RC Leiden, The Netherlands

**Keywords:** electroencephalography, migraine attack, prediction, cortical excitability, treatment

## Abstract

Migraine is a highly prevalent brain condition with paroxysmal changes in brain excitability believed to contribute to the initiation of an attack. The attacks and their unpredictability have a major impact on the lives of patients. Clinical management is hampered by a lack of reliable predictors for upcoming attacks, which may help in understanding pathophysiological mechanisms to identify new treatment targets that may be positioned between the acute and preventive possibilities that are currently available. So far, a large range of studies using conventional hospital-based EEG recordings have provided contradictory results, with indications of both cortical hyper- as well as hypo-excitability. These heterogeneous findings may largely be because most studies were cross-sectional in design, providing only a snapshot in time of a patient’s brain state without capturing day-to-day fluctuations. The scope of this narrative review is to (i) reflect on current knowledge on EEG changes in the context of migraine, the attack cycle, and underlying pathophysiology; (ii) consider the effects of migraine treatment on EEG features; (iii) outline challenges and opportunities in using EEG for monitoring attack susceptibility; and (iv) discuss future applications of EEG in home-based settings.

## 1. Introduction

Migraine attacks involve increased cortical excitability, which may thereby potentially serve as an early indicator of attack initiation [1,2,3]. Two main migraine subtypes are distinguished: migraine with aura (MA) and migraine without aura (MO). MA concerns about one-third of migraine patients and is characterized by patients reporting an aura preceding attacks, typically involving visual but sometimes also sensory disturbances [4]. For both MA and MO, hyperexcitability of the cortical areas has been implicated to contribute to attack susceptibility [3,5]. However, the processes which cause enhanced attack susceptibility remain largely unknown. This may also explain why preventive treatments, aimed at reducing cortical excitability, may be effective in only half of the patients treated [6]. Currently, there are no reliable indicators for changes in attack initiation, while such indicators would be highly relevant from both a research and clinical perspective [7]. This would allow for timely intervention and could reduce the heavy burden that the repeated occurrence of unpredictable attacks has on the well-being of patients [4,8].

Direct knowledge of changes in brain functioning throughout the migraine attack cycle has come from relatively recent fMRI imaging studies, which have underscored enhanced subcortical and cortical activity in the 48 h before the headache phase [9,10,11,12,13]. Whilst these studies have provided great insight into attack initiation mechanisms, fMRI measurements are very demanding for a patient and can only be performed in a hospital environment. The contrast of the hospital setting with a patient’s home environment is likely to influence attack susceptibility by increasing stress [14] or anticipation mechanisms in patients [15].

An alternative non-invasive tool to indicate changes in attack susceptibility could be electroencephalography (EEG). EEG measures the electrical activity in the brain using electrodes placed on the scalp. In contrast to brain imaging, EEG captures changes in the brain’s neuronal network activity with high temporal resolution. Traditionally, in ongoing EEG, various frequency bands (indicated in Hz) are identified including the alpha, beta, theta, and gamma bands. Each band is associated with specific underlying network processes that are affected by behavioral state and disease. Apart from spontaneous EEG, combining EEG with external stimuli such as sound, light, or odor allows for investigating the specific responsivity of, e.g., auditory, visual, or other sensory systems based on the so-called evoked potential [16].

Studies using EEG in migraine are mostly restricted to the research domain, as EEG does not have diagnostic value for migraine in the clinical setting [17], except when differentiating migraine from epilepsy, another paroxysmal brain disorder [1,17,18]. Nonetheless, EEG may provide an important contribution to the continuing demand for a migraine “biomarker” for both research and clinical purposes [7]. Various researchers have used standard clinical EEG recordings in this context, both in a resting state and in the context of sensory or other stimuli. Such studies have indicated increased EEG power spectral density, connectivity, and evoked potentials during the pre-ictal phase compared to the inter-ictal phase in migraine patients (as reviewed by [19,20,21,22]). The findings of these EEG studies are challenged by the lack of reproducibility of the identified EEG parameters that have been argued to involve blinding issues, the typical cross-sectional design, and the heterogeneity of disease status during measurements [3,23,24].

Recent technological advances have led to portable EEG devices that are small, user-friendly, and capable of capturing high-resolution and low-noise noise EEG signals [25,26,27]. This provides the opportunity to perform longitudinal EEG studies across the migraine cycle in a patient’s (home) environment, where patients undergo their natural changes in attack susceptibility.

In this narrative review, we discuss the potential of using EEG to identify changes in migraine attack susceptibility. After reflecting on the current knowledge on EEG changes in the context of migraine, attacks, and treatment effects, we will discuss the challenges faced with using standard clinical EEG for monitoring changes in attack susceptibility. Finally, we outline future applications of EEG in home-based settings. PubMed and earlier reviews on the use of EEG in migraine studies were used to identify relevant papers, with search terms including “EEG”, “encephalography”, “evoked responses”, “evoked potentials”, “VEP”, “excitability”, “ambulatory EEG”, “self-monitoring”, “migraine”, and “migraine treatment”, and their synonyms, guided by the scope indicated above. No specific exclusions were made in order to provide a broad overview of earlier studies, but since no systematic search strategy was used it is possible that not all EEG-related studies in the field of migraine were included.

## 2. Current Knowledge of Inter-Ictal EEG Changes in Migraine

Table 1 summarizes the findings of the sections below.

### 2.1. Changes in Spontaneous EEG in the Inter-Ictal Phase

Spontaneous EEG recordings (typically “resting-state” recordings during awake rest with eyes closed) in between attacks have revealed inconsistent findings for migraine patients compared to healthy controls, as well as MA compared to MO, as is detailed in earlier reviews [3,46,59]. Reported abnormalities for migraine compared to healthy controls include increased power in the delta [28], alpha, beta, and theta bands, interhemispheric alpha band asymmetries [19,29,30], and reduced alpha band coherence within the frontal cortex [35]. It should be noted that gamma band activity was not investigated in any of the studies. For the visual cortex, lower interhemispheric alpha coherence [32], lower alpha power [36], and increased beta band coherence [33] have been specifically associated with MA, suggesting that visual cortex plasticity changes underly aura susceptibility. Slowing in the alpha rhythm has also been specifically reported for MA [37].

To differentiate between MA and MO, functional connectivity analysis may have specific potential [77]. Both higher theta band connectivity (in the occipital cortex bilaterally) [31] and lower [34] theta band connectivity have been reported for MA. Together with findings of decreased [78], as well as increased connectivity in the beta band for MA [37], these connectivity studies suggest specific changes in resting state cortical network function in MA compared to MO [77,78]. By employing high-density EEG and analyzing data at sub-second intervals, researchers have identified distinct characteristics known as “microstates” that display variations between MA and MO across various brain regions, including extra-striate visual areas, the salience network, and dorsal attention networks [38].

### 2.2. Evoked Potential Changes in the Inter-Ictal Phase

The convergence of sensory and nociceptive pathways at the level of the thalamus [2] provides additional value to combining EEG with sensory stimuli, such as light, for recording sensory evoked potentials to monitor migraine-related changes in cortical responsivity [3,79]. In this context, visual evoked potentials (VEPs) have been used in various studies as an indirect readout of visual cortex responsivity in migraine patients compared to healthy controls during the inter-ictal phase. Some studies have reported VEP amplitudes to be unchanged [39] or reduced in the inter-ictal period [40,41]. Similarly, auditory response amplitudes were either unaltered [42] or reduced [43], while somatosensory response amplitudes were reduced [44,45] in patients compared to healthy controls. However, enhanced responsivity in between attacks has also been reported in various studies using visual [46,47,48], auditory [49,50], heat [51,52], affective picture [53,54,55], laser-evoked [56,57], or noxious stress stimuli [58]. When comparing MA with MO patients, VEP responses were either enhanced in MA [59,60,61,62] or unchanged [63,64] compared to MO.

In addition to single stimuli evoking cortical responses, repeated stimulation allows for the monitoring of migraine-related changes in cortical responsivity across multiple blocks of stimuli. Repetitive sensory stimuli presented in blocks, usually at frequencies around 3 Hz, can be used to study “habituation”, a feature occurring upon repeated stimulation in healthy subjects that is evident as a reduction in responsivity. An impaired habituation may reflect cortical hyper-responsivity. In migraine, impaired habituation has been reported for visual, auditory, somatosensory, nociceptive, and cognitive stimuli [23,50,65,66,67]. Since other studies have reported no change in habituation in migraine patients [24,39,68,69], also when comparing MA and MO [59], it is unclear whether a “lack of habituation” is a consistent feature in migraine [24,70,80]. Another phenomenon observable in EEG following repeated sensory stimulation is “photic drive”. This phenomenon relates to the EEG response to prolonged visual stimulation between 8 and 20 Hz [81] and was reported to be enhanced [70,71,72,73], attenuated [19], or variable [74] in migraine patients, suggesting complex network changes upon presentation of dynamic visual stimuli. MO patients were shown to display a stronger photic driving response than MA patients, suggesting a suppressed cortical function in MA contributing to a weaker response to photic stimulation [75]. One observation in another study showed that an increased coupling of visual cortical regions to other cortical areas was stronger in patients with longer inter-ictal periods, suggesting a modulation of this coupling by the state of the migraine cycle [72]. In addition to visual, auditory, or sensory stimuli, transcranial magnetic stimulation (TMS) has been used in absence of EEG recordings to indirectly investigate motor or visual cortex excitability during the inter-ictal phase [82,83,84,85], since the combination of TMS with EEG is technically challenging given TMS-induced artefacts. One study employed the combination to compare EEG phase clustering in response to TMS for patients with migraine and juvenile myoclonic epilepsy, and found no difference between the migraine and control groups, while phase clustering was enhanced in the epilepsy group [76].

## 3. Current Knowledge of EEG Changes during the Pre-Ictal, Aura, and Ictal Phases

Table 2 summarizes the findings of the sections below.

### 3.1. Resting State EEG Changes in Relation to Attacks

Based on cross-sectional measurements, a lower alpha band power and asymmetry of alpha power and the dominant alpha band frequency between hemispheres were reported during and before attacks without aura compared to the inter-ictal phase [86]. Nonetheless, before and after migraine attacks, EEG abnormalities have not been consistently identified [3,30]. In migraine with aura, alpha band asymmetry was demonstrated less than 48 h before the attack [88]. A recent study using high-density EEG identified decreased alpha power during the aura phase on a parieto–occipito–temporal location over the hemisphere contralateral to the visual aura, and this lasted into the headache phase [87]. The lack of slow potential changes during aura—indicative of the underlying phenomenon of cortical spreading depolarization (CSD)—may reflect the difficulty in detecting such changes through the intact scalp [94,95,96,97]. Also, auras may be caused by heterogeneous and spatially restricted CSD events that are directly visualized in the human cortex using intrinsic optical signals and cerebral blood flow monitoring [98,99]. If so, locally restricted CSDs might only cause local neuronal network depression in narrow parts of the cortex [94], which would not be detected by standard EEG. For the ictal headache phase, no differences during attacks have been found in EEG recordings that could discriminate MO from MA [30,46,89,90].

In contrast to cross-sectional studies, longitudinal studies have been able to identify some pre-ictal changes in spontaneous EEG features compared to the inter-ictal phase, and also between migraine patients and controls. These differences include EEG slowing, alpha and theta band asymmetry in occipito–parietal and temporal areas [22,89,90], enhanced EEG spectral power [20,22], and coherence [20,89,90]. An observation that EEG power and coherence were reduced during the inter-ictal phase compared to the pre-ictal phase was suggested to reflect a “normalization” of the brain network in the period before an attack [20].

### 3.2. Evoked Potential Changes in Relation to Attacks

By using pattern reversal VEPs in a longitudinal design, pre-ictal evoked amplitude features were found to be enhanced compared to inter-ictal recordings [60,69], particularly for high-contrast and spatial-frequency stimuli. This was discussed to possibly reflect a cyclic reduction in the intracortical inhibition of extra-striatal regions in the occipital cortex [60]. In addition to amplitude changes, a “lack of habituation” was reported for repeated inter-ictal evoked responses to visual and other stimulation modalities during attacks in some, but not all, studies [23,100]. Thus, whether cyclic changes occur due to habituation in the responses to repeated stimulations remains doubtful [70], in particular as longitudinal designs were only used in studies that did not observe an inter-ictal lack of habituation [51,60]. Nevertheless, with respect to visual cortex responsivity, the presence of transient alterations before attacks is supported by findings of attenuation in photic drive responses following an inter-ictal enhancement [89]. A related observation was found using a visual “chirp” stimulation paradigm (a repeated single-pulse flashing light stimulus with increasing frequency between 10 and 40 Hz at increments of 1 Hz) in a cross-sectional comparison of pre-ictal and inter-ictal recordings, which revealed increased responses in the beta band for recordings made during the pre-ictal phase [91].

Pre-ictal evoked EEG measures in migraine may also be measured using P300 peaks from a visual attention task. A study in which participants self-recorded their EEG at home while executing attention tasks revealed that increased power in the beta band, decreased power in the delta band, a reduction in the P300 amplitude, and reduced inter-trial coherence were found in pre-ictal recordings compared to inter-ictal recordings [92]. In line with this observation, another study described two female MO patients during three consecutive migraine cycles and attacks while somatosensory evoked potentials (SSEPs) were recorded daily, finding an increased level of pre-ictal somatosensory excitability [21]. Finally, fluctuations in sensorimotor processing during the migraine cycle were reported based on a difference in beta power between the pre- and inter-ictal phase, whereas no baseline differences were evident in comparison with controls [93].

Together, these studies indicate that changes in cortical responsivity during the pre-ictal phase can be detected using both resting state and evoked EEG paradigms. However, there is need for longitudinal study designs to reduce inter-individual variation. In addition to the typical cross-sectional designs used in most studies, the lack of reproducibility of the identified resting state and evoked EEG parameters for both inter-ictal and attack recordings could also be related to blinding issues, patient heterogeneity, and a possible heterogeneity of the disease state during measurements [3,23,24].

## 4. Migraine Treatment Effects on EEG

The effects of beta-blockers, anti-seizure medication, and monoclonal (receptor) antibodies (mAbs) targeting the calcitonin gene-related peptide (CGRP) or its receptor have been observed using EEG. These findings are of importance for several reasons: (1) they may demonstrate the potential central effects of prophylactics, reinforcing the understanding of migraine as a brain disease; (2) they provide a potential readout to evaluate treatment effects, which is particularly relevant to the introduction of new, more expensive treatment options, such as treatments blocking CGRP. Nonetheless, it remains uncertain which of the observed changes in EEG recording are the direct result of treatment or changes in the course of the disease, which is also modifiable by treatment.

Research on the effect of two prophylactic drug types, a calcium channel blocker (nifedipine) and two beta-blockers (propranolol and metoprolol), on VEPs has shown clinical improvements in reducing headache frequency related to a reduction in P100 amplitude but not latency. Interestingly, the P100 amplitude reverted to baseline levels in patients who did not experience a reduction in migraine days after prophylactic beta-blocker therapy, indicating a potential correlation between clinical response and neurophysiological changes [101]. Additionally, patients treated with various types of prophylactics, e.g., beta-blockers (types not indicated), anti-seizure medication (valproate), 5-HT_2_ receptor antagonists (pizotifen), anti-depressants (amitriptyline), or calcium channel blockers (flunarizine), showed restored P100 latencies compared to untreated patients, highlighting the impact of prophylactic medication on visual responsivity [101]. Finally, untreated MO patients showed a higher P100 latency compared to MA patients, suggesting different visual responsivity between MO and MA [102]. Taken together, different classes of prophylactics prove to have an impact on neurophysiological parameters by, for example, lowering or restoring amplitudes and latency times. However, these drug studies often have small sample sizes and lack longitudinal designs, limiting conclusions regarding clinical response and neurophysiological parameters.

The neurophysiological effects of anti-seizure medication have been demonstrated in various studies using VEPs. In a randomized controlled trial, MO patients were treated with topiramate, levetiracetam, or placebo, and EEG recordings in response to flashing light were analyzed before and after the start of treatment. Although both types of anti-seizure medication reduced migraine frequency, only levetiracetam reversed abnormal alpha band synchronization [103]. In another study, intravenous valproate administration during GTN-induced migraine headache showed direct changes in EEG spectral power and headache intensity, supporting the inhibitory role of anti-seizure medication on cortical excitability [104]. These findings suggest that anti-seizure medication might be effective by lowering cortical excitability through GABA-ergic and glutaminergic mechanisms, influencing alpha synchronization or spectral power.

The introduction of mAbs targeting calcitonin gene-related peptide (CGRP) or its receptor has provided a new class of prophylactic migraine treatments. Whether the effects of CGRP and CGRP receptor targeting medications act centrally is debated, since mAbs cannot cross the blood–brain barrier, but they have been suggested to instead exert action on the part of the trigeminovascular system (TGVS) in the lower brainstem [105]. Yet, EEG recordings before and after treatment with erenumab (mAb targeting CGRP receptor) have demonstrated a reduction in N1 and N2 amplitudes from trigeminal stimulation on the forehead after one month of treatment, possibly suggesting central changes as a result of the mAb treatment. Although clinical effectiveness was shown, no correlation existed between the lowered N1 or N2 amplitudes or the reduction in the number of headache days per month [106]. Additionally, galcanezumab treatment led to a reduction in EEG power after 3 months of treatment, which was measured using SSVEP from the occipital cortex at driving (5 Hz) and harmonic (10 and 15 Hz) frequencies [107]. The observed effects might be secondary central effects possibly explained by mAbs influencing the TGVS brainstem areas [108]. Differences in hypothalamic activation before and after galcanezumab or erenumab treatments demonstrated using MRI indeed suggest neurophysiological effects in deeper brain structures of the brain following treatment [109]. As a final point, it is important to highlight that publications on the neurophysiological effects of mAbs mainly included patients with chronic or high-frequency episodic migraine [83,106], which may limit the generalizability of the results. More longitudinal studies are needed to gain a better understanding of the neurophysiological effects of treatments that target CGRP.

## 5. Challenges in Using EEG for Monitoring Attack Susceptibility

A pressing issue in study designs involving EEG is the paroxysmal nature and the high inter- and intra-variability of migraine disease characteristics. So far, most EEG studies assessing attack susceptibility or changes in cortical responsivity have been clinic-based investigations that mainly use cross-sectional designs. Multiple study designs have been adapted to overcome the logistical problem behind data recording during the desired period of the migraine cycle but have so far yielded suboptimal results [19,93]. Examples of these approaches are continuous daily EEG recordings and retrospective migraine phase determination. Although both methodologies have proven their worth, they also possess disadvantages such as inaccurate migraine phase determination or a (disproportionately) high burden for patients, resulting in low participant numbers. Another challenge is that no gold standard exists for EEG studies on migraine, thus limiting comparability across studies with different designs and recording approaches. Guidelines have been formulated regarding basic setups, providing recording characteristics and stimulus capabilities for evoked potentials, but standardization has not been widely adopted so far [1,23,46]. Other design-related suggestions involve including blinding and randomization as critical tools to research pathophysiological and etiological pathways. Also, multi-modality studies are suggested, combining e.g., the temporal and spatial advantages of MRI and EEG [109]. Shortly, various considerations concerning the methodology of studies involving EEG in migraine have been put forward [23,46], but a more standardized approach remains a goal of the future [1,46,80].

The clinical setting of most EEG studies provides advantages in terms of controllability and safety, but it is also more demanding for participants and researchers to execute. For example, recordings in the lab require a patient to commute to and from the location. This inflexibility may result in psychological stress, which might influence cortical responsivity [14]. In addition, selection bias has been questioned in migraine studies [30] as a possible explanation for heterogeneous study results, and this may be partly explained by a limited inclusion of participants. Improved accessibility of measurements will increase inclusions and inclusiveness, allowing for the involvement of all migraine types when required.

## 6. Possibilities for Home-Based EEG Monitoring

By expanding beyond the lab, the limitations of clinic-based measurements may be overcome. While the use of home-based EEG has not been optimized, it may provide opportunities that deserve exploration, particularly when aiming to collect larger, more continuous datasets [110]. In recent years, new portable EEG devices have emerged on the market. Importantly, mobile EEG is accepted by patients [111], and both patients and technicians have labeled multiple current EEG devices as potentially very user-friendly in the domestic environment [25]. Despite differences in their features, several characteristics in their development are ubiquitous and form the basis for remote EEG data collection. In addition, increased battery life, at-home device rechargeability, and protected data storage options have largely improved [25,111]. Still, not all devices harbor wireless data acquisition [27], and the number of channels differs per device. Also, interesting novel approaches, such as an in-ear EEG device, are currently being evaluated, showing promising results and exhibiting high potential as a wearable device for easy home-based recordings [112]. All in all, the improvements in the basic features of these devices show promising options for EEG home monitoring. The actual selection of equipment will however depend on the question at hand or the preferred methodology (i.e., evoked potentials, resting state recordings, etc.) and, for this reason, validated scoring systems for the evaluation of different mobile EEG device characteristics may be useful [26].

In other clinical fields, such as in epilepsy, the use of EEG devices for home recordings has increased over the last decades [25,113]. EEG collection outside of the clinic has been well adopted to continuously monitor epilepsy patients at home, also within the context of optimizing treatment [113,114]. In the context of stroke, EEG devices outside of the lab can be used to diagnose large vessel occlusions in ambulances to optimize the flow of patients to a suitable healthcare institution [115,116].

As proof-of-concept investigation, a study on migraine patients demonstrated that simple-to-use EEG equipment can successfully be applied to home recordings [92]. In addition to the ability for longitudinal assessments of EEG changes in relation to the attack cycle, other advantages of home-based recordings for migraine include evaluations of treatment or lifestyle changes over long time periods [117] and enabling multimodality data collection [118].

EEG signal quality is of great importance to guaranteeing comprehensive data collection in the home environment outside the control of lab protocols. In this respect, studies on epilepsy show that recording high-quality data is possible, and that collected data are comparable to those collected in a hospital setting [25]. Nonetheless, the home-recording study in migraine patients indicated that due to artefacts in the EEG signals, half of the data proved to be unusable [92]. In this regard, quality evaluation during analysis is warranted to guarantee appropriate study results [119]. In addition, the use of headcaps has been previously debated in contrast to (traditional) individual gel-based electrode applications. Nonetheless, in-hospital data acquisition is currently regularly performed using various types of headcaps, such as gel- and water-based options. Moreover, the use of dry electrodes (pins or foam) has been introduced as an alternative. For home-based recordings, the type of cap chosen will depend on both signal quality and usability. For example, gel-based headcaps are not user-friendly in the sense that cleaning and maintaining the product is time-consuming, and this type of cap usually cannot be utilized without help [120]. In this sense, water-based and dry electrodes might be more suitable but may offer lower signal quality [119]. Nevertheless, emerging new products, such as pin electrodes coated with Ag-AgCl [121], enhance the possibilities for home-based recordings, but an evaluation of, for example, impedance and signal-to-noise ratios will be needed to evaluate data quality.

## 7. Conclusions and Outlook

In conclusion, studies that have utilized traditional hospital-based EEG recordings have produced conflicting results regarding the ability of EEG to monitor migraine attack susceptibility. The findings indicate both increased and decreased cortical excitability in the context of migraine in general and concerning the initiation of attacks. With respect to the latter, inconsistency across the studies may be largely attributed to the cross-sectional designs used with only a single recording timepoint being utilized, thus failing to account for daily fluctuations in brain activity. When implemented in a longitudinal design, EEG holds the potential for revolutionizing migraine research and treatment by providing insight into a patient’s brain activity changes that underlie the unpredictable occurrence of an attack. The various types of EEG paradigms and analyses and the possibility of utilizing ambulatory EEG devices offer novel avenues for data collection and analysis. Together, these can greatly enhance the overall understanding of the paroxysmal nature of migraine and the brain dynamics underlying attack initiation. The prospect of predicting attack susceptibility presents promising opportunities for effective migraine management that lie between the acute and preventive strategies that have been applied so far. As EEG technology continues to advance and make home-based applications possible, its integration into research and clinical practice is expected to lead to improved disease management.

## Figures and Tables

**Table 1:** Inter-ictal EEG changes in migraine.

Observation	Reference Number
Resting state	
Increased band power Delta Alpha Beta Theta bands	[28] [19] [29] [30]
Higher connectivity in the theta band range for the occipital cortex in MA	[31]
Lower interhemispheric alpha coherence and altered beta band activity in the bilateral occipital cortex in MA	[32,33]
Lower theta band connectivity in MA	[34]
Lower alpha band power and connectivity in the occipital cortex	[35,36,37]
“Microstate” abnormalities in multiple brain areas for MA vs. MO	[38]
Evoked potentials	
Reduced or unaltered responsivity Visual Unaltered Reduced Auditory Unaltered Reduced Somatosensory—reduced	[39] [40,41] [42] [43] [44,45]
Enhanced responsivity Visual Auditory Heat Affective picture Laser-evoked Noxious stress	[46,47,48] [49,50] [51,52] [53,54,55] [56,57] [58]
VEP responses between MA and MO Enhanced Unchanged	[59,60,61,62] [63,64]
Impaired habituation response to various stimuli Visual Auditory Somatosensory Nociceptive Cognitive	[23,65,66] [23,50,65] [23,67] [23,67] [23,67]
No change in habituation Migraine vs. controls MA vs. MO	[24,39,68,69] [59]
Photic drive response Enhanced (migraine vs. controls) Attenuated (migraine vs. controls) Variable Enhanced in MO vs. MA	[70,71,72,73] [19] [74] [75]
Transcranial magnetic stimulation	
Unaltered phase clustering	[76]

**Table 2:** EEG changes during the pre-ictal, aura, and ictal phases of migraine attacks.

Observation	Phase	Reference Number
Resting state		
Lower alpha band power and asymmetry of alpha rhythm and power in MO	Pre-ictal vs. inter-ictal	[86,87]
Alpha band asymmetry in MA < 48 h before the attack	Pre-ictal	[87,88]
EEG slowing, alpha and theta band asymmetry in occipito-parietal and temporal areas	Pre-ictal vs. inter-ictal	[22,89,90]
Enhanced EEG power	Pre-ictal vs. inter-ictal	[20,22]
Enhanced coherence	Pre-ictal vs. inter-ictal	[20,89,90]
No discriminatory observations during the ictal phase between MA and MO	Ictal phase	[29,46,89,90]
Evoked potentials		
Enhanced amplitude features following pattern reversal VEPs	Pre-ictal vs. inter-ictal	[60,69]
Lack of habituation during attacks (i) Ictal vs. inter-ictal (VEP, LEP, CNV, SSEPs) (ii) No interphasic differences (LEP, VEP)	-	[23,70] [51,60]
Increase in driving power after 12 Hz SSVEP	Pre-ictal vs. inter-ictal	[89]
Enhanced harmonic response to visual chirp stimulation	Pre-ictal vs. inter-ictal	[91]
Enhanced sensory evoked EEG measures in longitudinal designs (i) Reduced P300 (ii) Increased SSEPs (iii) Enhanced beta power (sensorimotor)	Pre-ictal vs. inter-ictal	[92] [21] [93]

## Data Availability

Not applicable.
